# Causal effect of blood osteocalcin on the risk of Alzheimer’s disease and the mediating role of energy metabolism

**DOI:** 10.1038/s41398-024-02924-w

**Published:** 2024-05-20

**Authors:** Xingzhi Guo, Yu-ying Yang, Rong Zhou, Ge Tian, Chang Shan, Jian-min Liu, Rui Li

**Affiliations:** 1 Department of Geriatric Neurology, Shaanxi Provincial People’s Hospital, Xi’an, 710068 Shaanxi China; 2 Xi’an Key Laboratory of Stem Cell and Regenerative Medicine, Institute of Medical Research, Northwestern Polytechnical University, Xi’an, 710072 Shaanxi China; 3 Department of Geriatric Neurology, the Third Affiliated Hospital of Xi’an Jiaotong University, Xi’an, 710068 Shaanxi China; 4 Department of Endocrine and Metabolic Diseases, Shanghai Institute of Endocrine and Metabolic Diseases, Ruijin Hospital, Shanghai Jiao Tong University School of Medicine, 200025 Shanghai, China; 5 Shanghai National Clinical Research Center for Metabolic Diseases, Key Laboratory for Endocrine and Metabolic Diseases of the National Health Commission of the PR China, Shanghai National Center for Translational Medicine, Ruijin Hospital, Shanghai Jiao Tong University School of Medicine, 200025 Shanghai, China; 6 Department of Endocrinology, Renji Hospital, Shanghai Jiao Tong University School of Medicine, 200127 Shanghai, China

**Keywords:** Predictive markers, Physiology

## Abstract

Growing evidence suggests an association between osteocalcin (OCN), a peptide derived from bone and involved in regulating glucose and lipid metabolism, and the risk of Alzheimer’s disease (AD). However, the causality of these associations and the underlying mechanisms remain uncertain. We utilized a Mendelian randomization (MR) approach to investigate the causal effects of blood OCN levels on AD and to assess the potential involvement of glucose and lipid metabolism. Independent instrumental variables strongly associated (*P* < 5E-08) with blood OCN levels were obtained from three independent genome-wide association studies (GWAS) on the human blood proteome (*N* = 3301 to 35,892). Two distinct summary statistics datasets on AD from the International Genomics of Alzheimer’s Project (IGAP, *N* = 63,926) and a recent study including familial-proxy AD patients (FPAD, *N* = 472,868) were used. Summary-level data for fasting glucose (FG), 2h-glucose post-challenge, fasting insulin, HbA1c, low-density lipoprotein cholesterol, high-density lipoprotein cholesterol, total cholesterol (TC), and triglycerides were incorporated to evaluate the potential role of glucose and lipid metabolism in mediating the impact of OCN on AD risk. Our findings consistently demonstrate a significantly negative correlation between genetically determined blood OCN levels and the risk of AD (IGAP: odds ratio [OR, 95%CI] = 0.83[0.72–0.96], *P* = 0.013; FPAD: OR = 0.81 [0.70–0.93], *P* = 0.002). Similar estimates with the same trend direction were obtained using other statistical approaches. Furthermore, employing multivariable MR analysis, we found that the causal relationship between OCN levels and AD was disappeared after adjustment of FG and TC (IGAP: OR = 0.97[0.80–1.17], *P* = 0.753; FPAD: OR = 0.98 [0.84–1.15], *P* = 0.831). There were no apparent instances of horizontal pleiotropy, and leave-one-out analysis showed good stability of the estimates. Our study provides evidence supporting a protective effect of blood OCN levels on AD, which is primarily mediated through regulating FG and TC levels. Further studies are warranted to elucidate the underlying physio-pathological mechanisms.

## Introduction

Alzheimer’s disease (AD) is the most prevalent neurodegenerative disorder, affecting more than 35 million individuals worldwide [1]. Osteoporosis, characterized by low bone mass and deterioration of bone quality, is a degenerative condition associated with an elevated risk of fractures and mortality [2]. Previous studies have revealed a bidirectional relationship between osteoporosis and AD, yet the underlying mechanisms remain elusive [3]. Recent evidence suggests that certain bone-derived factors, known as osteokines, play a role in regulating various physiological and pathophysiological processes, including brain development and cognitive function [4–6].

Among these osteokines, osteocalcin (OCN) is a hormone-like peptide primarily synthesized by osteoblasts responsible for bone formation and mineral density maintenance [7]. OCN also exerts significant effects on energy homeostasis, improving glucose and lipid metabolism, as well as on male fertility, muscle function, brain development, and cognitive function [8–11]. For instance, our recent research using a transgenic mouse model of AD demonstrated that OCN improved memory impairment of AD mice by promoting glycolysis in neuroglia [12]. Additionally, clinical studies have indicated that decreased blood OCN levels correlate with an increased risk of cognitive impairment and AD [13–15]. However, due to the limitations of traditional observational studies, such as susceptibility to confounding and reverse causation, the results have been inconsistent [13, 15], and the causal relationship between circulating OCN levels and AD remains unclear. Moreover, as abnormalities in glucose and lipid metabolism are closely associated with AD risk [16, 17], it is yet unknown whether the effects of OCN on AD are dependent on its role in regulating glucose and/or lipid metabolism.

Mendelian randomization (MR) is a powerful statistical approach that utilizes genetic variants as instrumental variables (IVs) to investigate causal relationships between different traits [18]. By capitalizing on the random allocation of genetic variants during conception, MR can provide robust evidence for causality while mitigating the biases introduced by confounding and reverse causation. The MR study design offers two main approaches: univariable MR (UVMR) and multivariable MR (MVMR). UVMR allows for the assessment of the causal association between a specific exposure and its corresponding outcomes, while MVMR enables the evaluation of potential mediators in these associations. In light of this, we employed both UVMR and MVMR methodologies to ascertain the causal relationship between blood OCN levels and the risk of AD, while also exploring the role of energy metabolism in this relationship.

## Methods

### Data source and study design

In this MR study, single nucleotide polymorphisms (SNPs) derived from summary statistics of genome-wide association studies (GWAS) were employed as instrumental variables (IVs). Summary-level data on blood OCN levels were obtained from three comprehensive GWAS on the human blood proteome of European descent, utilizing an aptamer-based approach known as the SOMAscan assay to measure the concentrations of human blood proteins. The sample sizes for the three GWAS from Eldjarn et al. [19], Gudjonsson et al. [20], and Sun et al. [21] were 35892, 5368, and 3301, respectively.

For the AD phenotype, summary statistics were obtained from a GWAS meta-analysis conducted by the International Genomics of Alzheimer’s Project (IGAP). This meta-analysis incorporated data from various consortia, including the Alzheimer Disease Genetics Consortium (ADGC), Cohorts for Heart and Aging Research in Genomic Epidemiology Consortium (CHARGE), European Alzheimer’s Disease Initiative (EADI), and Genetic and Environmental Risk in AD/Defining Genetic, Polygenic and Environmental Risk for Alzheimer’s Disease Consortium (GERAD/PERADES). The IGAP dataset comprised 21,982 AD cases and 41,944 controls of European descent [22]. Additionally, summary statistics from the latest GWAS analysis involving familial-proxy AD (FPAD) patients from the UK Biobank were utilized to further validate the MR results [23].

For the glucose metabolism phenotype, summary-level data on fasting glucose (FG, *N* = 200,622), post-challenge 2h-glucose (2h-Glu, *N* = 63,396), fasting insulin (FI, *N* = 151,013), and glycated hemoglobin (HbA1c, *N* = 200,622) were obtained from the Meta-Analysis of Glucose and Insulin-related Traits Consortium (MAGIC) [24]. Summary statistics for lipid metabolism traits, including low-density lipoprotein cholesterol (LDL-C), high-density lipoprotein cholesterol (HDL-C), total cholesterol (TC), and triglycerides (TG), were obtained from a meta-analysis of GWAS conducted by the Global Lipid Genetics Consortium (GLGC). To avoid potential bias introduced by sample overlapping, only the summary statistics excluding UK Biobank samples from the GLGC with up to 930,672 participants of European descent were used in this study [25]. For detailed information on the study design, please refer to the original publication (Supplementary Table S1). This study was performed using publicly available data, and no separate ethical approval was required.

### Instrumental variables selection and mendelian randomization analysis

**Fig. 1:**
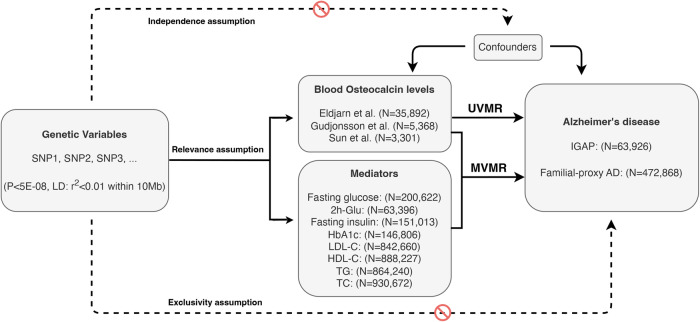
Flowchart and three assumptions to be satisfied in this Mendelian randomization study. The stop sign means genetic variables not associated with confounding factors and AD. Relevance assumption indicates that IVs are strongly associated with blood osteocalcin levels, independence assumption indicates that IVs are not associated with confounding factors, and exclusivity assumption indicates that IVs affect the risk of AD via blood osteocalcin levels directly. AD Alzheimer’s disease, SNP single nucleotide polymorphism, LD linkage disequilibrium, 2h-Glu 2h-glucose post-challenge, HbA1c glycated hemoglobin, HDL-C high-density lipoprotein cholesterol, LDL-C low-density lipoprotein cholesterol, TG triglycerides, TC total cholesterol, UVMR univariable Mendelian randomization, MVMR multivariable Mendelian randomization, IGAP International Genomics of Alzheimer’s Project, N number .

To ensure the validity of our MR analysis, three key assumptions must be satisfied. First is the relevance assumption, which requires IVs to be strongly associated with blood OCN levels. Second is the independence assumption, which states that IVs should not be associated with any confounding factors. Lastly, the exclusivity assumption suggests that IVs should directly affect the risk of AD through blood OCN (Fig. 1). To meet these MR assumptions, we selected SNPs that surpassed the genome-wide significance threshold (*P* < 5E–08) as IVs. These SNPs were then clumped based on the linkage disequilibrium (LD) structure from the 1000 Genomes Project, with a threshold of r^2^ < 0.01 within 10 Mb for individuals of European descent. In cases where a corresponding outcome had missing SNPs, we substituted them with overlapping proxy SNPs that exhibited complete LD (r^2^ = 1). Additionally, we also calculated the F-statistic value for each IVs using the formula (β/SE)^2^ [26]. Meanwhile, the Steiger filtering test was applied, and only those SNPs with a higher explanatory variance in the exposure than the outcome was retained [27]. For MVMR analysis, conditional F-statistics were computed to evaluate the strength of the genetic instruments after conditioning on other exposures in the model [28]. This MR study was conducted in compliance with the strengthening the reporting of observational studies in epidemiology using MR (STROBE-MR) guideline [29, 30].

The random-effect inverse variance weighted (IVW) model served as the primary approach for calculating causal estimates. To assess the stability of the MR results, we conducted six sensitivity analyses, including MR-Egger, weighted median, weighted mode, simple median, maximum likelihood, and MR-Pleiotropy RESidual Sum and Outlier (MR-PRESSO) tests. The MR-Egger regression intercept was used to examine the presence of horizontal pleiotropy in the MR analysis. The MR-PRESSO method enabled the identification of outliers and provided a causal estimate without their influence. We also performed leave-one-out analysis to evaluate the stability of the MR estimates, and Cochran’s Q statistic was utilized to assess heterogeneity. For the MVMR analysis, we iteratively combined the IVs for blood OCN levels from Eldjarn et al. and glucose metabolism (FG, 2h-Glu, FI, HbA1c) as well as lipid metabolism (LDL-C, HDL-C, TC, TG) as previously described [31]. After that, we included those putative mediators (*P* < 0.05) in the same MVMR model to further assess their role in mediating the relationship between blood OCN and AD risk. Three statistical methods, namely IVW, MR-Egger, and Lasso, were employed to calculate the MVMR estimates. For multiple comparisons adjustment, a P-value less than 0.017 (0.05/3 exposures) were considered as statistical significance, while a P-value less than 0.05 as suggestive significance. To perform the statistical analyses and generate plots, we utilized the following software packages: TwoSampleMR (V-0.5.6), MR-PRESSO (V-1.0), MendelianRandomization (V-0.7.0), MVMR (V-0.4.0), and forestploter (V-0.2.3) in R software (V-4.2.2) [28, 32–34].

## Results

After harmonizing exposure and outcome effects, there were ten, two, and one valid IVs for blood OCN levels from Eldjarn et al., Gudjonsson et al., and Sun et al., respectively. For the ten IVs from Eldjarn et al. two of them were missing in AD GWAS from IGAP and only eight IVs were available for MR analysis using the IGAP dataset. The F-statistic values for the MR study ranged from 30.03 to 448.98, indicating no weak instrument bias. Detailed information on each IV is provided in Table 1.

**Table 1:** Effect sizes of single nucleotide polymorphism associated with blood osteocalcin levels (r^2^ < 0.01).

SNP	CHR	BP^a^	EA	NEA	EAF	BETA	SE	*P*-VALUE	*N*	F-statistic	Gene	PMID
rs1831272	1	196766611	G	A	0.182	−0.057	0.010	6.71E-09	35667	33.62	Near *CHFR3*	37794188
rs185320691	6	32522515	C	G	0.094	0.087	0.013	9.70E-11	35768	41.88	*HLA-DRB5*	37794188
rs2019727	1	196705584	A	T	0.165	0.107	0.010	4.31E-25	35667	107.06	*CFH*	37794188
rs241430	6	32835043	C	T	0.597	0.050	0.008	1.39E-10	35767	41.18	*TAP2*	37794188
rs3132469	6	31488790	G	A	0.810	0.120	0.010	1.29E-34	35768	150.57	*MICB-DT*	37794188
rs3830076	6	32128467	T	C	0.091	0.085	0.013	2.25E-10	35772	40.24	*ATF6B/FKBPL*	37794188
rs61803031	1	161632464	T	C	0.182	−0.055	0.010	3.37E-08	35689	30.48	*FCGR3B*	37794188
rs76359966	6	32487803	G	T	0.659	0.136	0.007	3.48E-77	35768	345.77	Near *HLA-DRB9*	37794188
rs9263708	6	31127493	C	T	0.191	0.084	0.010	8.28E-18	35771	73.88	*PSORS1C1*	37794188
rs9273429	6	32659679	A	G	0.562	0.164	0.008	1.18E-99	35768	448.98	*HLA-DQB1*	37794188
rs3117116	6	32399240	A	G	0.775	0.209	0.023	6.59E-20	5368	84.10	*BTNL2/TSBP1-AS1*	35078996
rs35617250	1	196710552	T	C	0.167	0.166	0.026	8.73E-11	5368	42.26	*CFH*	35078996
rs71631868	1	196846581	C	T	NA	0.168	0.031	4.27E-08	3301	30.03	*CHFR2*	29875488

*SNP* single nucleotide polymorphism, *CHR* Chromosome, *BP* Base position, *EA* Effect allele, *NEA* Non-effect allele, *SE* Standard error, *N* Number, *NA* Not available.

^a^based on GRCh38.

**Fig. 2:**
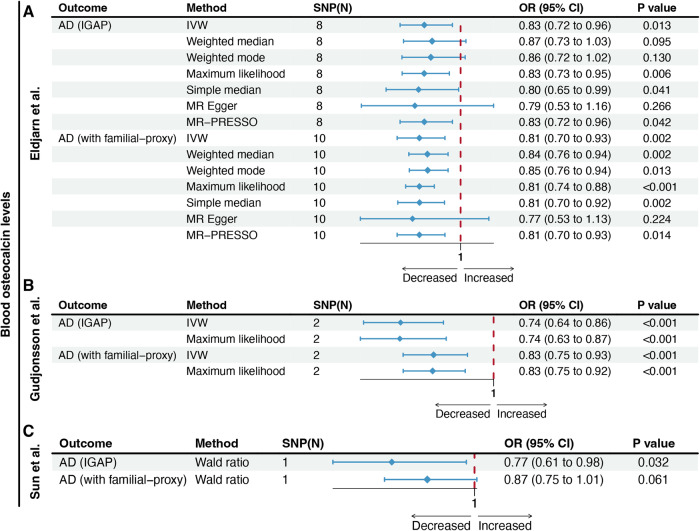
Effects of blood osteocalcin levels on the risk of Alzheimer’s disease. Using two different summary-level data on AD, genetically predicted blood osteocalcin levels were associated with a decreased risk of AD via seven different statistical approaches. **A**–**C** Showed the MR estimates using summary-level data on blood osteocalcin from Eldjarn et al. (**A**), Gudjonsson et al. (**B**), and Sun et al. (**C**), respectively. AD Alzheimer’s disease, IVW inverse variance weighted, SNP single nucleotide polymorphism, MR Mendelian randomization, MR-PRESSO MRPleiotropy RESidual Sum and Outlier, OR odds ratio, IGAP International Genomics of Alzheimer’s Project, N number.

**Fig. 3:**
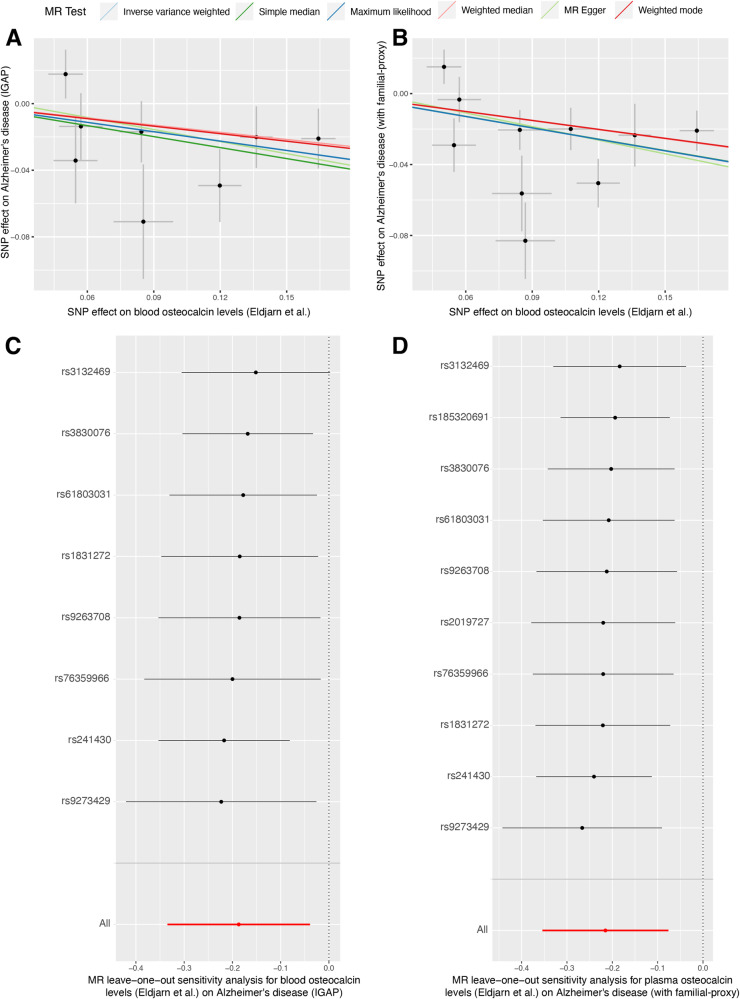
Scatter plots and leave-one-out plots for the causal association between blood osteocalcin levels and Alzheimer’s disease. **A**, **B** Showed the SNPs’ effect on blood osteocalcin levels (Eldjarn et al.) and AD using GWAS summary-level data from IGAP and study with familial-proxy AD. The slope indicated the causal estimates for each method. **C**, **D** Showed the leave-one-out plots for the causal effect of blood osteocalcin levels on AD. AD Alzheimer’s disease, IVW inverse-variance weighted, MR Mendelian randomization, SNP single nucleotide polymorphism, IGAP International Genomics of Alzheimer’s Project.

Using the IVW method and summary statistics from Eldjarn et al., the UVMR results demonstrated a negative association between genetically determined blood OCN levels and the risk of AD (IGAP: odds ratio [OR] = 0.83, 95% confidence interval [CI] = 0.72–0.96, *P* = 0.013; FPAD: OR = 0.81, 95%CI = 0.70–0.93, *P* = 0.002) (Fig. 2A). This association was further confirmed using summary-level data from Gudjonsson et al. and Sun et al. (Fig. 2B, C). The results obtained from other sensitivity analysis approaches showed consistent trends, although not all of them reached statistical significance (Fig. 2). The MR-Egger regression intercept test indicated no apparent horizontal pleiotropy. The Cochran Q statistic suggested potential heterogeneity in the AD-proxy dataset but not in the IGAP datasets (Supplementary Table S2). There were two potential outliers (rs185320691 and rs241430) were identified in the MR-PRESSO test using the AD-proxy dataset, but the results consistently showed an inverse association between OCN levels and AD risk after correcting the outliers (OR = 0.82, 95%CI = 0.73–0.93, *P* = 0.013). Leave-one-out analysis did not reveal any significant single SNP driving the bias of estimates, indicating robust results (Fig. 3). In addition, to explore the biological relevance of OCN in AD, we further used IVs within gene regions involved in bone homeostasis to validate the effect of OCN on AD. Four SNPs, including rs1831272, rs2019727, rs3830076, and rs61803031 previously reported to be associated with genes suggestively linked to bone homeostasis were used [35–40] (Table 1). The results consistently suggested an inverse relationship between OCN levels and AD risk (IGAP: OR = 0.59, 95%CI = 0.38–0.93, *P* = 0.022; FPAD: OR = 0.77, 95%CI = 0.62–0.96, *P* = 0.019) (Supplementary Fig. S1).

Using the IVW approach and summary statistics from Eldjarn et al. in the MVMR analysis, the results indicated a significant and consistent association between blood OCN levels and a reduced risk of AD after adjusting for 2h-Glu (OR = 0.81, 95%CI = 0.72–0.91, *P* < 0.001), FI (OR = 0.80, 95%CI = 0.71–0.91, *P* < 0.001), HbA1c (OR = 0.83, 95%CI = 0.72–0.96, *P* = 0.009), HDL-C (OR = 0.83, 95%CI = 0.75–0.93, *P* < 0.001), LDL-C (OR = 0.85, 95%CI = 0.76–0.96, *P* = 0.007), and TG (OR = 0.87, 95%CI = 0.77–0.97, *P* = 0.013). These associations were further validated in the FPAD dataset, as shown in Table 2. However, using the IGAP dataset, the causal association between blood OCN levels and AD risk disappeared after adjusting for FG (OR = 0.88, 95%CI = 0.76–1.02, *P* = 0.087) or TC (OR = 0.89, 95%CI = 0.78–1.01, *P* = 0.066), but not well replicated in the FPAD dataset with MR-Egger and Lasso methods (Table 2). The conditional F-statistics were larger than 10 for blood OCN levels and glucose profiles (2h-Glu, FI, HbA1c, and FG), but less than 10 for lipid profiles (HDL-C, LDL-C, TG, and TC), indicating that weak instrument bias may occur in the MVMR analysis for OCN and lipid profiles (Table 2).

**Table 2:** MVMR results of blood osteocalcin on the risk of AD after adjustment of each mediator.

Exposure	Mediators	Method	AD (IGAP)				AD (with familial-proxy)			
			SNP (*N*)	OR(95%CI)	*P*	Conditional F-statistics	SNP (*N*)	OR(95%CI)	*P*	Conditional F statistics
**Blood osteocalcin levels**^**a**^	**2h-Glu**	IVW	23	0.81 (0.72–0.91)	<0.001	31.48	24	0.81 (0.72–0.91)	<0.001	31.48
		Egger	23	0.82 (0.72–0.92)	0.001		24	0.81 (0.71–0.92)	0.001	
		Lasso	23	0.81 (0.72–0.92)	<0.001		24	0.81 (0.72–0.90)	<0.001	
	**FG**	IVW	102	0.88 (0.76–1.02)	0.087	13.83	105	0.86 (0.77–0.96)	0.008	13.45
		Egger	102	0.80 (0.67–0.94)	0.008		105	0.86 (0.77–0.96)	0.007	
		Lasso	102	0.85 (0.75–0.96)	0.010		105	0.87 (0.80–0.95)	0.002	
	**FI**	IVW	52	0.80 (0.71–0.91)	<0.001	22.06	54	0.81 (0.73–0.91)	<0.001	21.56
		Egger	52	0.83 (0.71–0.95)	0.009		54	0.83 (0.74–0.93)	0.001	
		Lasso	52	0.81 (0.72–0.91)	<0.001		54	0.83 (0.76–0.91)	<0.001	
	**HbA1c**	IVW	95	0.83 (0.72–0.96)	0.009	15.26	99	0.83 (0.74–0.92)	<0.001	14.69
		Egger	95	0.83 (0.72–0.96)	0.009		99	0.83 (0.75–0.92)	<0.001	
		Lasso	95	0.82 (0.73–0.92)	<0.001		99	0.81 (0.75–0.88)	<0.001	
	**HDL-C**	IVW	798	0.83 (0.75–0.93)	<0.001	2.65	844	0.83 (0.77–0.89)	<0.001	2.59
		Egger	797	0.83 (0.73–0.94)	0.004		843	0.83 (0.77–0.90)	<0.001	
		Lasso	797	0.82 (0.75–0.89)	<0.001		843	0.85 (0.80–0.90)	<0.001	
	**LDL-C**	IVW	554	0.85 (0.76–0.96)	0.007	7.94	577	0.85 (0.78–0.92)	<0.001	7.48
		Egger	552	0.85 (0.74–0.97)	0.020		575	0.85 (0.78–0.92)	<0.001	
		Lasso	552	0.85 (0.77–0.94)	0.001		575	0.90 (0.84–0.96)	0.002	
	**TG**	IVW	642	0.87 (0.77–0.97)	0.013	2.78	680	0.89 (0.82–0.96)	0.003	2.70
		Egger	642	0.82 (0.71–0.94)	0.005		680	0.86 (0.78–0.94)	0.002	
		Lasso	642	0.84 (0.76–0.93)	<0.001		680	0.89 (0.84–0.95)	<0.001	
	**TC**	IVW	713	0.89 (0.78–1.01)	0.066	1.99	748	0.89 (0.82–0.98)	0.016	1.96
		Egger	713	0.87 (0.73–1.03)	0.098		748	0.90 (0.82–0.98)	0.021	
		Lasso	713	0.84 (0.76–0.94)	0.002		748	0.94 (0.87–1.01)	0.090	

*MVMR* Multivariable Mendelian randomization, *IGAP* International Genomics of Alzheimer’s Project, *IVW* Inverse variance weighted, *AD* Alzheimer’s disease, *2h-Glu* 2h-glucose post-challenge, *FG* Fasting glucose, *FI* fasting insulin, *HbA1c* Glycated hemoglobin, *HDL-C* High-density lipoprotein cholesterol, *LDL-C* Low-density lipoprotein cholesterol, *TG* Triglycerides; *TC* Total cholesterol, *P* P-value, *N* Number, *SNP* Single nucleotide polymorphism, *OR* Odds ratio, *CI* Confidence interval.

^a^summary-level data from Eldjarn et al.

To further assess the combined impact of FG and TC in mediating the effect of OCN on AD, a MVMR was performed by putting FG and TC in the same MVMR model. Using the IVW approach, the MR estimates showed that the protective effect of OCN on AD was disappeared after adjusting for FG and TC at the same time dataset (IGAP: OR = 0.97, 95%CI = 0.80–1.17, *P* = 0.753; FPAD: OR = 0.98, 95%CI = 0.84–1.15, *P* = 0.831). The MVMR estimates obtained from the MR-Egger and Lasso methods were consistent with the IVW results (Table 3), indicating good stability. The conditional F-statistics were larger than 10 for blood OCN levels in MVMR analysis after conditioning on both FG and TC (Table 3).

**Table 3:** MVMR results of blood osteocalcin on the risk of AD after adjustment of FG and TC.

Exposures	Method	AD (IGAP)				AD (with familial-proxy)			
		SNP (*N*)	OR(95%CI)	*P*	Conditional F statistics	SNP (*N*)	OR(95%CI)	*P*	Conditional F statistics
**Osteocalcin levels**^**a**^ **(FG** and **TC)**	IVW	798	0.97(0.80–1.17)	0.753	32.24	839	0.98(0.84–1.15)	0.831	32.37
	MR-Egger	790	0.97(0.81–1.18)	0.790		831	0.98(0.84–1.15)	0.821	
	Lasso	790	0.89(0.80–0.99)	0.032		831	0.96(0.89–1.03)	0.230	

*MVMR* Multivariable Mendelian randomization, *IGAP* International Genomics of Alzheimer’s Project, *IVW* Inverse variance weighted, *AD* Alzheimer’s disease, *FG* Fasting glucose, *TC* Total cholesterol, *P* P-value, *N* Number, *SNP* Single nucleotide polymorphism, *OR* Odds ratio, *CI* Confidence interval.

^a^based on summary-level data from Eldjarn et al.

## Discussion

The association between OCN, cognitive function, and AD has been a subject of significant research interest over the past decade, revealing some observed associations. In this MR study, utilizing summary statistics from two different AD studies, we further strengthen the evidence by demonstrating a causal relationship between genetically predicted blood OCN levels and a decreased risk of AD. These findings suggest a protective role of OCN in the development of AD. Furthermore, our MVMR analysis results indicate that the protective effect of OCN on AD may primarily rely on its regulation of FG and TC levels.

OCN, a crucial peptide derived from osteoblasts, is known to play a role in bone remodeling and is closely linked to bone mineral density (BMD) [41, 42]. Clinical studies have shown that individuals with osteopenia or osteoporosis are at a higher risk of developing AD [43, 44]. Moreover, lower blood OCN levels have been associated with brain microstructural changes and poorer cognitive function in elderly adults [13, 45]. Consistent with these findings, our MR analysis demonstrates an inverse relationship between genetically determined blood OCN levels and the risk of AD. Additionally, a recent animal study from our research group has shown that OCN can improve cognitive impairment in an AD transgenic mouse model (APP/PS1 mice) [12]. However, it is worth noting that some studies have suggested increased blood OCN levels in AD patients [15, 46]. It remains unclear whether this is a compensatory response of the bone to combat underlying diseases, similar to the situations observed in obesity and diabetes [47, 48], or if there are other unidentified mechanisms.

Dysfunction in both glucose metabolism and lipid metabolism has been consistently associated with an increased risk of AD [16, 17, 49]. For example, patients with Type 2 diabetes (T2D) or elevated levels of TC and LDL-C were at a higher risk of AD [50, 51]. A cohort study reported an association between early-onset AD and higher levels of LDL-C [52]. Similarly, a systematic review involving nearly 6500 AD patients revealed elevated LDL-C levels in individuals with AD [53]. Another meta-analysis by Liu and colleagues with up to 5948 individuals also showed that blood TC and LDL-C levels were tightly associated with mild cognitive impairment and AD [50]. Pathologically, increased blood levels of LDL-C, TC, TG, and decreased levels of HDL-C have been linked to an accumulation of β-amyloid plaques in the hippocampus and adjacent temporal lobe of AD patients [54]. Furthermore, previous MR studies have demonstrated a positive association between blood FG, TC and LDL-C levels and the risk of AD [55, 56], while lowering blood glucose and LDL-C levels has shown a causal effect in reducing the risk of AD [57, 58].

To evaluate the role of glucose and lipid metabolism in the causal relationship between OCN and AD, we conducted an MVMR analysis. Our findings indicate that the inverse association between blood OCN levels and AD risk is diminished after adjusting for FG and TC, suggesting that OCN may ameliorate AD through its regulation of both FG and TC. Indeed, population studies have shown a negative correlation between serum OCN levels, FG, and TC [59]. Our previous meta-analysis involving 23,381 participants also revealed a negative correlation between blood levels of OCN and FG and HbA1c [60]. Moreover, animal studies have further revealed that OCN treatment significantly reduces serum FG and TC levels in both diabetic and non-diabetic rats [61].

The underlying mechanism behind the beneficial role of OCN in reducing the risk of AD through the amelioration of glucose and lipid metabolism, particularly by lowering FG and TC levels, is complex. There are reports linking dysregulated lipid metabolism to AD. For example, elevated cholesterol levels within the lipid rafts of neuron cell membranes can enhance the activity of key enzymes involved in amyloid protein precursor (APP) cleavage, such as β-secretase/β-site amyloid precursor protein cleavage enzyme-1 (BACE-1) and γ-secretase, leading to increased production of β-amyloid [62–64]. Additionally, the neurotoxic cholesterol oxidation product 27-hydroxycholesterol has been implicated in the pathogenesis of AD [65]. Therefore, regulating lipid metabolism may be an important approach to reduce the development of AD. It is worth noting that OCN stimulates the release of adiponectin, an important adipokine that can have a beneficial effect on lipoprotein metabolism, including TC reduction [66]. Meanwhile, our MVMR results revealed that the causal effect of OCN on AD was also partially dependent of glucose metabolism, especially for FG. Numerous studies have showed that impaired FG was related to increased cerebral beta-amyloid accumulation and atrophy, and associated with a higher risk of AD [67, 68]. It is reported that the levels of glucose transporter-3 in AD patients were decreased, leading to impaired glycolytic flux, which was related to the severity of AD pathology [69]. In contrast, studies in both humans and animals have showed that blood osteocalcin levels were associated with improved glucose metabolism and insulin sensitivity [60, 70, 71]. Furthermore, in our recent mouse study, we discovered that OCN can improve cognitive defects in AD mice by promoting glycolysis in neuroglia [12]. Taken together, these findings suggest that both glucose and lipid metabolism may play an essential mediating role in the causal pathway between OCN and AD.

## Limitations

There are several limitations to consider in this study. Firstly, we employed a relatively relaxed r^2^ threshold (=0.01) to select a sufficient number of instrumental variables (IVs) for MR analysis, which may have led to an overestimation of the causal association. Secondly, although the MR estimates from different approaches showed consistent trends, some of them did not reach statistical significance, possibly due to the small sample size for blood OCN levels. Thirdly, the Cochran Q test revealed potential heterogeneity. However, the results of MR-PRESSO test consistently showed an inverse association between OCN levels and AD after correcting the outliers. Fourthly, despite the genetic instruments strongly predicting blood OCN levels in the UVMR analysis, the MVMR analysis may remain susceptible to bias due to conditionally weak instruments, diminishing the power of MVMR in estimating causal effects. Thus, the potential mediating influence of energy metabolism, especially lipid profiles, on the causal pathways linking blood OCN levels with AD in our study requires further confirmation. Finally, the findings of this study were based on individuals of European descent, and further validation in other racial/ethnic groups is needed.

## Conclusion

In summary, this MR study provides evidence that elevated blood OCN levels are associated with a decreased risk of AD through the regulation of FG and TC, indicating a potential beneficial role of OCN in preventing AD. However, it is essential to conduct additional studies in both human populations and animal models to verify these causal associations and fully elucidate the underlying mechanisms.

### Supplementary information

Supplementary Tables and Figure

STROBE-MR-checklist

## Data Availability

The GWAS summary statistics for blood osteocalcin levels, AD, glucose metabolism, and lipid metabolism were from the original articles.

## Code availability

All R scripts applied in this MR study are available from the authors upon reasonable request.
