# Supplementary material for: Causal effect of blood osteocalcin on the risk of Alzheimer’s disease and the mediating role of energy metabolism
Source: Transl Psychiatry. 2024 May 20;14:205. doi: 10.1038/s41398-024-02924-w (PMC11106250; doi:10.1038/s41398-024-02924-w)
Supplement: Supplementary file 1 — Supplementary Tables and Figure [file 41398_2024_2924_MOESM1_ESM.docx]

**Supplementary Table S1.** Summary of the GWAS datasets used in this Mendelian randomization study.

| **Phenotype** | **Author/Consortium** | **Year** | **Sample size (N)** | **SNP(N)** | **PMID** |
| --- | --- | --- | --- | --- | --- |
| **Exposure** |  |  |  |  |  |
| Blood OCN levels | Eldjarn et al. | 2023 | 35,892 | 33,452,791 | 37794188 |
|  | Gudjonsson et al. | 2022 | 5,368 | 7,505,327 | 35078996 |
|  | Sun et al. | 2018 | 3,301 | 10,534,735 | 29875488 |
| 2h-Glu | MAGIC | 2021 | 63,396 | 30,098,704 | 34059833 |
| FG | MAGIC | 2021 | 200,622 | 34,064,006 | 34059833 |
| FI | MAGIC | 2021 | 151,013 | 32,635,792 | 34059833 |
| HbA1c | MAGIC | 2021 | 146,806 | 33,811,879 | 34059833 |
| LDL-C | GLGC | 2022 | 842,660 | 36,864,579 | 34887591 |
| HDL-C | GLGC | 2022 | 888,227 | 36,588,494 | 34887591 |
| TC | GLGC | 2022 | 930,672 | 36,871,085 | 34887591 |
| TG | GLGC | 2022 | 864,240 | 37,005,452 | 34887591 |
| **Outcome** |  |  |  |  |  |
| AD | IGAP | 2019 | 63,926 | 10,528,610 | 30820047 |
| AD with familial-proxy | Schwartzentruber et al. | 2021 | 472,868 | 10,602,762 | 33589840 |

Note: GWAS, Genome-wide association study; IGAP, International Genomics of Alzheimer's Project; AD, Alzheimer’s disease; OCN, osteocalcin; 2h-Glu, 2h-glucose post-challenge; FG, fasting glucose; FI, fasting insulin; HbA1c, glycated hemoglobin; HDL-C, high-density lipoprotein cholesterol; LDL-C, low-density lipoprotein cholesterol; TG, triglycerides; TC, total cholesterol; N, number; SNP, single nucleotide polymorphism; N, number.

**Supplementary Table S2.** Heterogeneity and pleiotropy test in univariable Mendelian randomization analysis.

| **Exposure** | **Outcome** | **SNP (N)** | **Cochran Q test** | | **MR-Egger Intercept (P)** | **MR-PRESSO RSSobs (P)** |
| --- | --- | --- | --- | --- | --- | --- |
|  |  |  | **MR-Egger (P)** | **IVW (P)** |  |  |
| Osteocalcin (Eldjarn et al.) | AD (IGAP) | 8 | 8.58 (0.198) | 8.72 (0.274) | 0.01 (0.770) | 10.34 (0.365) |
| Osteocalcin (Eldjarn et al.) | AD (with familial-proxy) | 10 | 25.93 (0.001) | 26.10 (0.002) | 0.00 (0.825) | 31.21 (0.01) |
| Osteocalcin (Gudjonsson et al.) | AD (IGAP) | 2 | - | 0.40 (0.526) | - | - |
| Osteocalcin (Gudjonsson et al.) | AD (with familial-proxy) | 2 | - | 1.25 (0.264) | - | - |
| Osteocalcin (Sun et al.) | AD (IGAP) | 1 | - | - | - | - |
| Osteocalcin (Sun et al.) | AD (with familial-proxy) | 1 | - | - | - | - |

Note: IGAP, International Genomics of Alzheimer's Project; AD, Alzheimer’s disease; IVW, Inverse variance weighted; P, P-value; N, number.


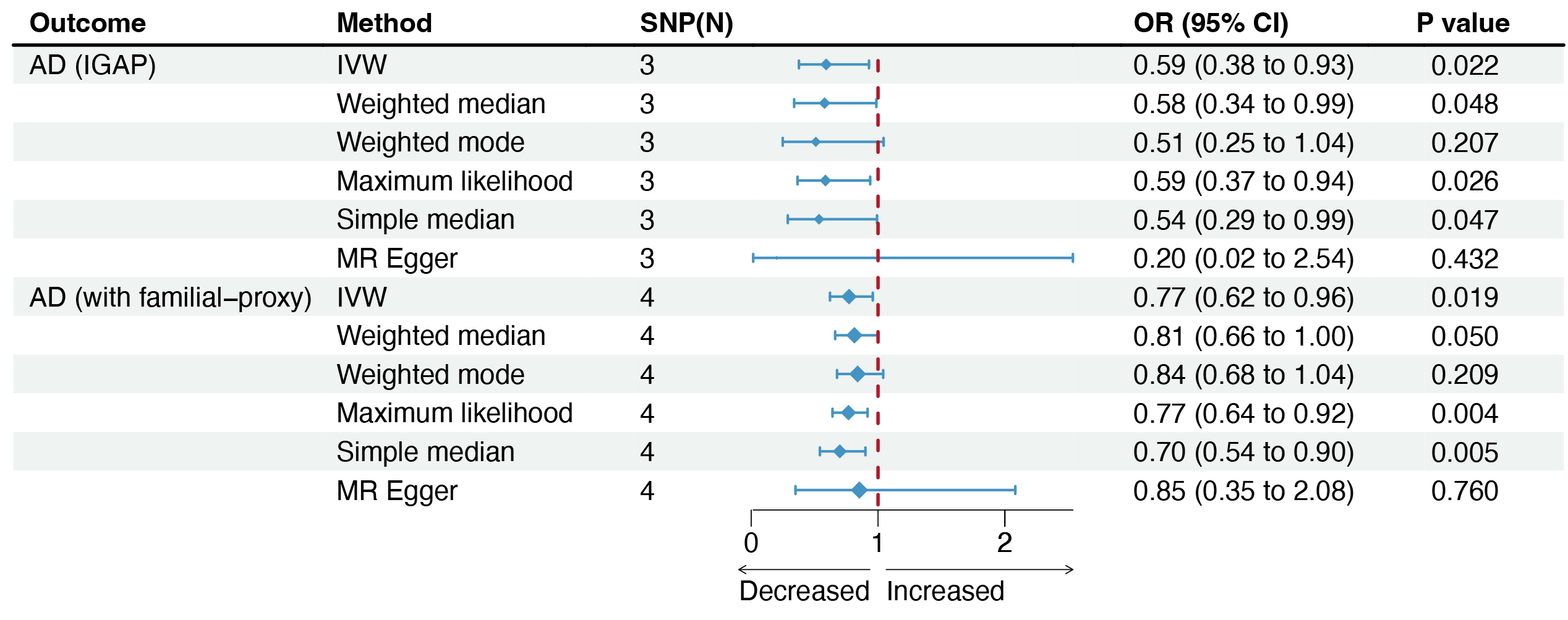


**Supplementary Figure S1.** Effects of blood osteocalcin levels on the risk of Alzheimer’s disease using IVs associated with bone homeostasis affecting osteocalcin levels. Using IVW method with four IVs (rs1831272, rs2019727, rs3830076, and rs61803031), genetically predicted blood osteocalcin levels (Eldjarn et al.) remained associated with a decreased risk of AD, which was further confirmed by other statistical approaches. AD, Alzheimer’s disease; IVW, inverse variance weighted; SNP, single nucleotide polymorphism; OR, odds ratio; IGAP, International Genomics of Alzheimer's Project; N, number.
