# Supplementary material for: Causal effect of blood osteocalcin on the risk of Alzheimer’s disease and the mediating role of energy metabolism
Source: Transl Psychiatry. 2024 May 20;14:205. doi: 10.1038/s41398-024-02924-w (PMC11106250; doi:10.1038/s41398-024-02924-w)
Supplement: Supplementary file 2 — STROBE-MR-checklist [file 41398_2024_2924_MOESM2_ESM.docx]

**STROBE-MR checklist of recommended items to address in reports of Mendelian randomization studies**^1^ ^2^

| **Item No.** | **Section** | **Checklist item** | **Page No.** | **Relevant text from manuscript** |
| --- | --- | --- | --- | --- |
| 1 | **TITLE and ABSTRACT** | Indicate Mendelian randomization (MR) as the study’s design in the title and/or the abstract if that is a main purpose of the study | 3 | “We utilized a Mendelian randomization (MR) approach ...” |
|  | **INTRODUCTION** |  |  |  |
| 2 | **Background** | Explain the scientific background and rationale for the reported study. What is the exposure? Is a potential causal relationship between exposure and outcome plausible? Justify why MR is a helpful method to address the study question | 6 | “By capitalizing on the random allocation of genetic variants during conception, MR can provide robust evidence for causality while mitigating the biases introduced by confounding and reverse causation ” |
| 3 | **Objectives** | State specific objectives clearly, including pre-specified causal hypotheses (if any). State that MR is a method that, under specific assumptions, intends to estimate causal effects | 6-7 | “we employed both UVMR and MVMR methodologies to ascertain the causal relationship between blood OCN levels and the risk of AD, while also exploring the role of energy metabolism in this relationship” |
|  | **METHODS** |  |  |  |
| 4 | **Study design and data sources** | Present key elements of the study design early in the article. Consider including a table listing sources of data for all phases of the study. For each data source contributing to the analysis, describe the following: |  |  |
|  | a) | Setting: Describe the study design and the underlying population, if possible. Describe the setting, locations, and relevant dates, including periods of recruitment, exposure, follow-up, and data collection, when available. | 6 | “...blood OCN levels were obtained from three comprehensive GWAS on the human blood proteome of European descent” |
|  | b) | Participants: Give the eligibility criteria, and the sources and methods of selection of participants. Report the sample size, and whether any power or sample size calculations were carried out prior to the main analysis | 6 | “The sample sizes for the three GWAS from Eldjarn et al [19], Gudjonsson et al. [20], and Sun et al. [21] were 35892, 5368, and 3301, respectively.” |
|  | c) | Describe measurement, quality control and selection of genetic variants | 8 | “To ensure the validity of our MR analysis, three key assumptions must be satisfied. First ..” |
|  | d) | For each exposure, outcome, and other relevant variables, describe methods of assessment and diagnostic criteria for diseases | 6 | “utilizing an aptamer-based approach known as the SOMAscan assay to measure the concentrations of human blood proteins” |
|  | e) | Provide details of ethics committee approval and participant informed consent, if relevant | 8 | “This study was performed using publicly available data, and no separate ethical approval was required” |
| 5 | **Assumptions** | Explicitly state the three core IV assumptions for the main analysis (relevance, independence and exclusion restriction) as well assumptions for any additional or sensitivity analysis | 8 | “To meet these MR assumptions, we selected SNPs that surpassed...” |
| 6 | **Statistical methods: main analysis** | Describe statistical methods and statistics used |  |  |
|  | a) | Describe how quantitative variables were handled in the analyses (i.e., scale, units, model) | 9 | “The random-effect inverse variance weighted (IVW) model served as the primary approach for calculating causal estimates” |
|  | b) | Describe how genetic variants were handled in the analyses and, if applicable, how their weights were selected | 8 | “we also calculated the F-statistic value for each IVs using” |
|  | c) | Describe the MR estimator (e.g. two-stage least squares, Wald ratio) and related statistics. Detail the included covariates and, in case of two-sample MR, whether the same covariate set was used for adjustment in the two samples | 9 | “The random-effect inverse variance weighted (IVW) model served as...” |
|  | d) | Explain how missing data were addressed | 8 | “we substituted them with overlapping proxy SNPs that exhibited complete LD” |
|  | e) | If applicable, indicate how multiple testing was addressed | 10 | “For multiple comparisons adjustment, a P-value less than 0.017 (0.05/3 exposures) were considered as statistical significance...” |
| 7 | **Assessment of assumptions** | Describe any methods or prior knowledge used to assess the assumptions or justify their validity | 9 | “The MR-Egger regression intercept ...” |
| 8 | **Sensitivity analyses and additional analyses** | Describe any sensitivity analyses or additional analyses performed (e.g. comparison of effect estimates from different approaches, independent replication, bias analytic techniques, validation of instruments, simulations) | 9 | “To assess the stability of the MR results, we conducted six sensitivity analyses...” |
| 9 | **Software and pre-registration** |  |  |  |
|  | a) | Name statistical software and package(s), including version and settings used | 10 | “TwoSampleMR (V-0.5.6), MR-PRESSO (V-1.0), MendelianRandomization (V-0.7.0)...” |
|  | b) | State whether the study protocol and details were pre-registered (as well as when and where) | NA | NA |
|  | **RESULTS** |  |  |  |
| 10 | **Descriptive data** |  |  |  |
|  | a) | Report the numbers of individuals at each stage of included studies and reasons for exclusion. Consider use of a flow diagram | 10 | “After harmonizing exposure and outcome effects...” |
|  | b) | Report summary statistics for phenotypic exposure(s), outcome(s), and other relevant variables (e.g. means, SDs, proportions) |  | NA |
|  | c) | If the data sources include meta-analyses of previous studies, provide the assessments of heterogeneity across these studies |  | NA |
|  | d) | For two-sample MR:  i.  Provide justification of the similarity of the genetic variant-exposure associations between the exposure and outcome samples  ii.  Provide information on the number of individuals who overlap between the exposure and outcome studies | 10 | “there were ten, two, and one valid IVs for blood OCN levels from Eldjarn et al., Gudjonsson et al., and Sun et al, respectively” |
| 11 | **Main results** |  |  |  |
|  | a) | Report the associations between genetic variant and exposure, and between genetic variant and outcome, preferably on an interpretable scale | 10 | “Table 1” |
|  | b) | Report MR estimates of the relationship between exposure and outcome, and the measures of uncertainty from the MR analysis, on an interpretable scale, such as odds ratio or relative risk per SD difference | 10-12 | “genetically determined blood OCN levels and the risk of AD...” |
|  | c) | If relevant, consider translating estimates of relative risk into absolute risk for a meaningful time period |  | NA |
|  | d) | Consider plots to visualize results (e.g. forest plot, scatterplot of associations between genetic variants and outcome versus between genetic variants and exposure) | 11 | “Figure 3...” |
| 12 | **Assessment of assumptions** |  |  |  |
|  | a) | Report the assessment of the validity of the assumptions | 11 | “MR-Egger regression intercept test indicated no apparent horizontal pleiotropy” |
|  | b) | Report any additional statistics (e.g., assessments of heterogeneity across genetic variants, such as *I^2^*, Q statistic or E-value) | 11 | “The Cochran Q statistic suggested potential heterogeneity...” |
| 13 | **Sensitivity analyses and additional analyses** |  |  |  |
|  | a) | Report any sensitivity analyses to assess the robustness of the main results to violations of the assumptions | 11-12 | “The MR-Egger regression intercept test indicated no apparent horizontal pleiotropy....” |
|  | b) | Report results from other sensitivity analyses or additional analyses | 10-11 | “The results obtained from other sensitivity analysis approaches showed consistent trends ...” |
|  | c) | Report any assessment of direction of causal relationship (e.g., bidirectional MR) |  | NA |
|  | d) | When relevant, report and compare with estimates from non-MR analyses |  | NA |
|  | e) | Consider additional plots to visualize results (e.g., leave-one-out analyses) | 11 | “Leave-one-out analysis did not reveal any significant single SNP...” |
|  | **DISCUSSION** |  |  |  |
| 14 | **Key results** | Summarize key results with reference to study objectives | 13 | “These findings suggest a protective role of OCN in the development and progression of ...” |
| 15 | **Limitations** | Discuss limitations of the study, taking into account the validity of the IV assumptions, other sources of potential bias, and imprecision. Discuss both direction and magnitude of any potential bias and any efforts to address them | 16-17 | “There are several limitations to consider in this study...” |
| 16 | **Interpretation** |  |  |  |
|  | a) | Meaning: Give a cautious overall interpretation of results in the context of their limitations and in comparison with other studies | 13-16 | 2nd-5th paragraph in the Discussion |
|  | b) | Mechanism: Discuss underlying biological mechanisms that could drive a potential causal relationship between the investigated exposure and the outcome, and whether the gene-environment equivalence assumption is reasonable. Use causal language carefully, clarifying that IV estimates may provide causal effects only under certain assumptions | 13-16 | 2nd-5th paragraph in the Discussion |
|  | c) | Clinical relevance: Discuss whether the results have clinical or public policy relevance, and to what extent they inform effect sizes of possible interventions | 13-14 | 2nd paragraph in the Discussion |
| 17 | **Generalizability** | Discuss the generalizability of the study results (a) to other populations, (b) across other exposure periods/timings, and (c) across other levels of exposure | 17 | “the findings of this study were based on individuals of European descent...” |
|  | **OTHER INFORMATION** |  |  |  |
| 18 | **Funding** | Describe sources of funding and the role of funders in the present study and, if applicable, sources of funding for the databases and original study or studies on which the present study is based | 18 | “Funding statement: ...” |
| 19 | **Data and data sharing** | Provide the data used to perform all analyses or report where and how the data can be accessed, and reference these sources in the article. Provide the statistical code needed to reproduce the results in the article, or report whether the code is publicly accessible and if so, where | 18 | “Availability of data and materials: The GWAS summary statistics...” |
| 20 | **Conflicts of Interest** | All authors should declare all potential conflicts of interest | 18 | “Conflict of interest: None” |

This checklist is copyrighted by the Equator Network under the Creative Commons Attribution 3.0 Unported (CC BY 3.0) license.

1. Skrivankova VW, Richmond RC, Woolf BAR, Yarmolinsky J, Davies NM, Swanson SA, et al. Strengthening the Reporting of Observational Studies in Epidemiology using Mendelian Randomization (STROBE-MR) Statement. JAMA. 2021;under review.

2. Skrivankova VW, Richmond RC, Woolf BAR, Davies NM, Swanson SA, VanderWeele TJ, et al. Strengthening the Reporting of Observational Studies in Epidemiology using Mendelian Randomisation (STROBE-MR): Explanation and Elaboration. BMJ. 2021;375:n2233.
